# Prospective longitudinal study: use of faecal gluten immunogenic peptides to monitor children diagnosed with coeliac disease during transition to a gluten‐free diet

**DOI:** 10.1111/apt.15277

**Published:** 2019-05-10

**Authors:** Isabel Comino, Verónica Segura, Luis Ortigosa, Beatríz Espín, Gemma Castillejo, José Antonio Garrote, Carlos Sierra, Antonio Millán, Carmen Ribes‐Koninckx, Enriqueta Román, Alfonso Rodríguez‐Herrera, Jacobo Díaz, Jocelyn Anne Silvester, Ángel Cebolla, Carolina Sousa

**Affiliations:** ^1^ Sevilla Spain; ^2^ Tenerife Spain; ^3^ Reus Spain; ^4^ Valladolid Spain; ^5^ Málaga Spain; ^6^ Valencia Spain; ^7^ Madrid Spain; ^8^ Ceuta Spain; ^9^ Winnipeg Canada; ^10^ Boston Massachusetts

## Abstract

**Background:**

Treatment for coeliac disease is a lifelong strict gluten‐free diet. Although guidelines recommend regular follow‐up with dietary interviews and coeliac serology, these methods may be inaccurate.

**Aim:**

To evaluate the usefulness of faecal gluten immunogenic peptides to support the diagnosis and to determine the adherence to the gluten‐free diet in coeliac children.

**Methods:**

Multicentre prospective observational study including 64 coeliac children. Faecal gluten peptides, and tissue transglutaminase and deamidated gliadin peptide antibodies were analyzed at diagnosis, and 6, 12 and 24 months thereafter. Gluten consumption was estimated from gluten peptide levels.

**Results:**

Most children (97%) had detectable gluten peptides at diagnosis. On a gluten‐free diet, the rate of gluten peptides increased from 13% at 6 months to 25% at 24 months. Mean estimated gluten exposure dropped from 5543 mg/d at diagnosis to 144 mg/d at 6 months, then increased to 606 mg/d by 24 months. In contrast, deamidated gliadin peptide antibodies normalised and only 20% had elevated tissue transglutaminase antibody by 24 months. The elevation of tissue transglutaminase antibody was more prolonged in patients with detectable gluten peptides (*P *<* *0.05). Nevertheless, absolute levels of tissue transglutaminase antibody had low sensitivity to identify patients with detectable gluten peptides (*P *>* *0.1). Dietitian assessment was only moderately correlated with gluten peptide detection (κ = 0.5).

**Conclusions:**

Faecal gluten peptides testing may guide treatment of coeliac disease prior to diagnosis and during the assessment diet adherence. Further studies could determine if early identification of gluten exposure reduces the need for expensive/invasive investigations for non‐responsive coeliac disease. ClinicalTrials.gov Number: NCT02711397.

## 1 INTRODUCTION

Coeliac disease is an immune‐mediated disorder of the small intestine that affects ~1% of most populations.^1^, ^2^ This lifelong condition is initiated by exposure to dietary gluten in genetically susceptible individuals and can affect any organ or tissue. Consequently, a variety of gastrointestinal and extra‐intestinal symptoms are observed, such as abdominal pain, malabsorption, anaemia, failure to thrive, osteoporosis and, occasionally, lymphoma.^3^

A lifelong gluten‐free diet (GFD) is currently the only available treatment for coeliac disease and has been shown to improve quality of life substantially, both in patients initially presenting with gastrointestinal symptoms and in asymptomatic patients.^4^ However, adherence to the GFD is not easy because gluten is ubiquitous and often not explicitly listed as an ingredient in food products. As a result, persistent symptoms and enteropathy are common among coeliac patients who are trying to follow a GFD. Specifically, research shows that 25%‐40% of adults with coeliac disease have persistent enteropathy after 2 years on a GFD.^5^, ^6^, ^7^ Children are thought to recover more quickly, and data suggests that 5%‐19% of coeliac children on a GFD may have persistent enteropathy despite treatment with a GFD for at least 1 year.^8^, ^9^, ^10^, ^11^, ^12^, ^13^ Moreover, while the experts agree strict adherence to the GFD is crucial for the health of coeliac patients, there are no evidence‐based recommendations regarding the most efficient way to assess GFD adherence.^14^ Typically, dietary history, symptoms and serum antibody tests are used to assess non‐adherence. However, a detailed dietary history is time consuming and requires the collaboration of a dietician, so in routine clinical practice, physicians often rely upon the patient's own subjective assessment of dietary adherence, or the presence of adverse symptoms to guide dietician referral. The difficulty with this approach is that many non‐adherent patients may be asymptomatic. In addition, if patients do have symptoms, these may be as a result of coexisting disorders, such as irritable bowel syndrome, microscopic colitis or pancreatic insufficiency.^15^ Concerningly, although coeliac antibody tests are widely used for routine monitoring of coeliac patients, these tests were never approved for this purpose and have low sensitivity in detection of persistent mucosal lesion in coeliac patients on a GFD.^16^ Therefore, none of these methods offer an accurate measure of dietary adherence. Whilst duodenal biopsy is considered the ‘gold standard’ for assessment of celiac disease activity,^17^ certain associations, such as the European Society for Pediatric Gastroenterology, Hepatology and Nutrition (ESPGHAN) Coeliac Disease working group strongly advises against regular re‐biopsy in children on a GFD, due to unnecessary risks for the patient, negative impacts on quality of life, and pointless high medical costs.^18^

Monoclonal antibodies capable of sensitively and specifically detecting gluten immunogenic peptides (GIP) have been used to develop assays for detecting inadvertent gluten consumption by measuring GIP in human samples (faeces and urine). These assays may be a practical method to assess dietary adherence in coeliac patients.^19^, ^20^, ^21^ GIP are resistant to gastrointestinal digestion and account for immunogenic reactions in T cells of patients with coeliac disease.^22^ Unlike traditional methods to monitor GFD adherence which only evaluate the consequences of GFD transgressions, this non‐invasive method enables a direct and quantitative assessment of gluten exposure. A recent study demonstrated the clinical usefulness of GIP as a marker of GFD adherence in patients following a GFD for at least 1 year.^20^ Although it is well known that compliance with the GFD overall decreases on long term follow‐up, however strict adherence to a GFD in the first year after the diagnosis crucial to favour mucosal recovery. For this reason, the aim of the present study was to evaluate the measurement of GIP in stools as a marker of GFD adherence in newly diagnosed coeliac children in a prospective multicentre clinical trial. Furthermore, this was compared to traditional methods used to assess GFD adherence: dietitian review of four‐day food recrod and coeliac serology (tissue transglutaminase, tTG and deamidated gliadin peptide antibodies, DGP).

## 2 MATERIALS AND METHODS

### 2.1 Study design

A multicentre prospective observational study of detection of GIP in stool in a cohort of children newly diagnosed with coeliac disease was performed at seven Spanish secondary and tertiary referral hospitals: Hospital Nuestra Señora de la Candelaria (Tenerife), Hospital Regional Carlos Haya (Málaga), Hospital Universitario y Politécnico La Fe (Valencia), Hospital Universitario Virgen del Rocío (Sevilla), Hospital Universitario Virgen de Valme (Sevilla), Instituto Hispalense de Pediatría (Sevilla), Hospital Universitario Río Hortega (Valladolid) and Hospital Sant Joan de Reus (Tarragona). There were four study visits at diagnosis and, 6 (from 4‐9 months), 12 (from 10‐15 months) and 24 (from 16‐24 months) months after diagnosis. The first visit (diagnosis) was at the time of diagnostic endoscopy when patients were untreated (diet with gluten). All participants were instructed to follow a GFD by clinical dieticians with expertise in coeliac disease. Samples of faeces and blood were collected at each study visit and participants completed a four‐day food record according to the dietician instructions. The study protocol was reviewed by the ethics committee at each participating hospital and written informed consent was obtained from the parents or legal guardians.

### 2.2 Study population

Children (less than 18 years of age) with active coeliac disease were recruited at time of diagnostic endoscopy. Inclusion criteria were: (a) symptoms—either gastrointestinal (eg, abdominal pain, diarrhea, constipation, weightloss, flatulence, bloating, vomiting, lack of appetite) or atypical (e.g., iron deficiency anaemia, chronic fatigue, behavioural changes, poor growth); (b) elevated serum EMA IgA, tTG IgA/IgG and/or DGP IgA/IgG; (c) HLADQ2 and/or HLADQ8 genotype; and, (d) small intestinal histology consistent with coeliac disease (Marsh II‐III). Exclusion criteria were: (a) history of kidney, liver or severe psychiatric disease; (b) seizure disorder and/or current use of anticonvulsants; (c) use of any antibiotics within the year prior to enrolment.

All subject data were recorded in an electronic data capture (EDC) system, including: age, weight, height, intestinal histology, symptoms, comorbiditions, clinical test results, adverse events, family history of coeliac disease, date of coeliac disease diagnosis, adherence to GFD, and details sample collection and visit attendance.

### 2.3 Faeces and blood collection

Subjects were instructed to collect 2‐4 g stool in a sealed container after recording their food intake for 4 days. Specimens were dropped‐off within 24 hours of collection and were kept at −20°C at all times until processing.

Blood samples were collected in two 3 mL vacutainer tubes with EDTA‐K3 anticoagulant and centrifuged at 2000 *g* within 30 minutes of collection to obtain plasma which was stored at −80°C until analysis. Investigators performing stool and serum analysis were blinded to GFD status at the time of sample collection.

### 2.4 Quantification of GIP in stool samples

Stool GIP concentration was determined by sandwich enzyme‐linked immunosorbent assay (ELISA; iVYDAL In Vitro Diagnostics iVYLISA GIP Stool kit, Biomedal S.L., Seville, Spain) according to the manufacturer's protocol. Briefly, stool samples were mixed with 9 ml Universal Gluten Extraction Solution (UGES; Biomedal S.L., Seville, Spain) per gram of stool then incubated at 50°C for 60 minutes with gentle agitation to release the GIP from the stool matrix. After extraction, samples were diluted 1:10 with dilution solution and ELISA was performed using the provided G12 coated microtiter plate, standards (50, 25, 6.25, 3.13, 1.56 ng/ml GIP) and positive and negative controls. Thus, results were expressed as μg GIP per gram faeces. Each sample was run in duplicate and at least two different aliquots of each sample were tested on different days.

The validity of this method in detecting GFD transgressions was determined by the analytical sensitivity (limits of detection and quantification 0.06 and 0.16 μg GIP per gram faeces, respectively) and the diagnostic sensitivity and specificity (98.5% and 100%, respectively).^20^

### 2.5 Estimation of gluten consumption

A calibration factor allowed estimation of the ingestion of gluten in coeliac patients from stool measurements. Specifically, the equation for estimating daily gluten consumption in milligrams (y variable) based upon faecal GIP concentration (in micrograms per gram) (x variable) was determined from measured mean values of 6.2 and 14.9 μg GIP per gram faeces during controlled gluten challenges of 9 and 30 grams per day.^19^, ^20^, ^23^ Fitting to a second‐order polynomial going through the origin gave the relation y = 0.0649x^2^ + 1.0461x.

### 2.6 Serology

The levels of tTG IgA and DGP IgA antibodies (IgG in IgA‐deficient patients) were determined by ELISA using the EliA^TM^ Celikey^®^ and EliA^TM^ Gliadin kits, respectively, according to the manufacturer's protocol (Phadia, Freiburg, Germany). Measurements were performed in duplicate and the results expressed as U/ml. The manufacturer recommended cut‐off of >10 U/mL was used.

### 2.7 Dietary questionnaire

To assess gluten exposure, a structured food questionnaire of 27 items was administered to record the foods consumed during the 4 days prior to stool and blood collection. The food items were classified into eight predefined groups: dairy (milk and cheese); complex carbohydrates (bread, cereals, pasta, rice, potato, legumes, and nuts); meats (red meat, fish, cold cuts, and eggs); fruits (whole or juiced); vegetables; fats (vegetable oils, butter, and cream); sweetened beverages (sodas, bottled juices, and energy drinks); and other (baked goods, candy, snacks, etc.). Images of standard portion sizes were included as a guideline for portion quantification. Subjects were asked to record the amount and type of food consumed, brand, time of meal, and if it was labelled as gluten‐free. They were also asked to note if they were aware of having consumed any gluten‐containing foods.

### 2.8 Statistical analysis

Quantitiative variables are expressed as median with interquartile range (IQR), and the categorical variables as percentages. Goodness‐of‐fit to normal was checked using the Shapiro‐Wilk test. Pearson's chi‐squared test was used for categorical variables, and the chi‐squared test for ordinal variables. Statistical analysis of the degree of concordance of the dichotomously evaluated diagnostic techniques was performed using Cohen's kappa index (κ), following the criteria of Landis and Koch^24^ for the qualitative interpretation of the strength of concordance. The Mann‐Whitney *U* test was used to compare quantitative variables in independent groups. For all cases, *P* < 0.05 was considered statistically significant. Data analysis was performed with SPSS 23.0 for Windows (SPSS Inc).

## 3 RESULTS

The study population consisted of 64 children (21 males and 43 females) with a median age of 4 years (IQR 1.5‐9 years; Table 1). Participant retention was 94% at 6 months, 78% at 12 months and 55% at 24 months (the most common reason for loss to follow‐up were: moving out of the study area, not attending follow‐up visits and forgetting to collect samples).

**Table 1:** Characteristics of 64 patients with celiac disease enrolled in the study

Characteristic	Patients, n (%)
Sex	
Male	21 (33%)
Female	43 (67%)
Age (y)	
<2	16 (25%)
2‐6	21 (33%)
7‐18	27 (42%)
Median age	4
Interquartile range (P_25_‐P_75_)	1.5‐9

### 3.1 Detection of GIP in stool

**Figure 1:**
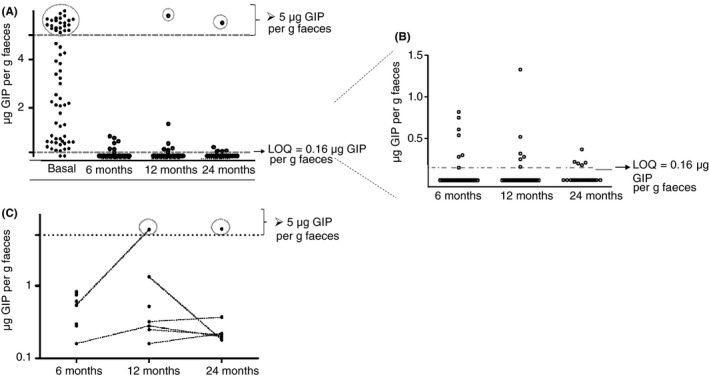
Concentration of gluten immunogenic peptides (GIP) in stools of patients with newly diagnosed coeliac disease during monitoring of the gluten‐free diet. (A) Levels of faecal GIP at the basal and follow‐up visits (basal, 6, 12 and 24 mo). (B) Levels of faecal GIP at 6, 12 and 24 mo. (C) Levels of GIP in transgressing patients in the different follow‐up visits (log scale). GIP, gluten immunogenic peptides; LOQ, limit of quantification

At the initial visit, before starting the GFD, during the expected gluten challenge for diagnosis, 62 (97%) patients had detectable GIP in the provided stool sample. After diagnosis and treatment with a GFD, 13 (23%) of patients had detectable levels of GIP in at least one visit, whereas 11 (20%) were noncompliant according to the questionnaire. Overall, 11% of all coeliac patients were noncompliant by both methods, 48% were gluten free by both methods (κ = 0.5) and 39% were discordant. In general, GFD adherence rates declined as the study progressed (Figure 1A,B). The rate of GIP positive stools was 13% at 6 months, 18% at 12 months and 25% at 24 months. Notably, 46% of transgressors had detectable GIP at two or more follow‐up visits (Figure 1C).

**Figure 2:**
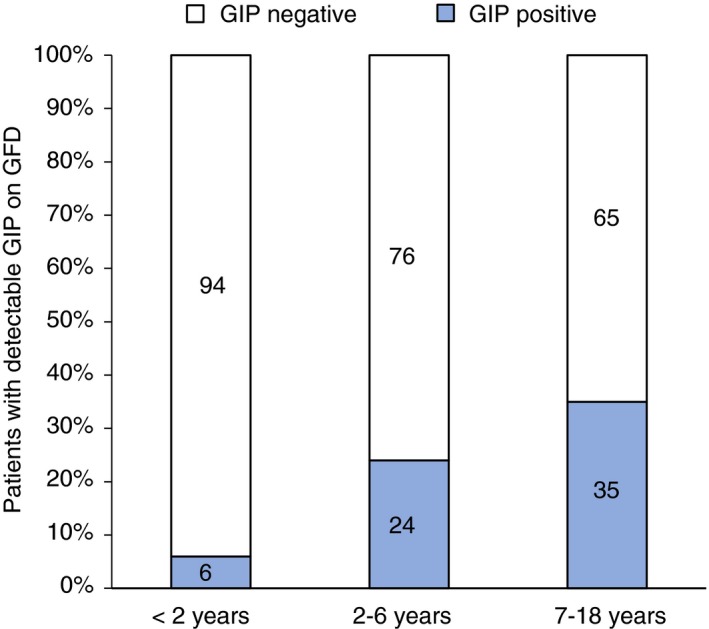
Gluten immunogenic peptides detection on a GFD according to patient age. Percentage distribution of stools collected during a GFD with detectable GIP according to age. GIP, gluten immunogenic peptides. GFD, gluten‐free diet

The rate of GIP positive stools during follow‐up increased with age (*P *=* *0.041). Specifically, GIP were detected in stools from 6% of children with coeliac disease before 2 years of age, and in 24% and 35% in those diagnosed at 2‐6 years and at an older age, 7‐18 years, respectively (Figure 2).

Eleven participants (17%) reported no classic symptoms prior to diagnosis with coeliac disease and instead were biopsied for atypical symptoms or laboratory abnormalities. The rate of dietary transgressions in this group was 33% compared to 19% for the group diagnosed due to classical coeliac disease symptoms (*P = *0.57).

### 3.2 Estimated gluten ingestion

An estimate of gluten consumption by coeliac patients following a GFD was determined by measuring GIP in stool. Both the mean and median values for each visit are reported due to the non‐normal distribution of gluten ingestion (Table 2). At diagnosis, estimated daily gluten consumption was 5543 mg (mean) with a 95% CI [4345‐6741 mg] and 3882 mg (median). During transition to a GFD, we found 342 mg gluten (mean), 95% CI [67‐616 mg] and 104 mg (median) in samples tested. There was a trend toward increased gluten consumption during the follow‐up period. After diagnosis, estimated gluten exposure was (mean/median) 144/99 mg/d at 6 months, 452/105 mg/d at 12 months, and 606/117 mg/d at 24 months.

**Table 2:** Estimated gluten consumption based upon GIP measured in stool^a^

GFD duration (mo)	n	GIP concentration (μg/g stool)			Estimated gluten consumption (mg/d)		
		Mean	Median	IQR	Mean	Median	IQR
0 (diagnosis)	64	3.82	3.11	0.60‐7.60	5543	3882	691‐11 699
6	54	0.14	0.09	0.05‐0.14	144	99	49‐149
12	39	0.34	0.10	0.05‐0.15	452	105	52‐160
24	24	0.43	0.11	0.06‐0.18	606	117	58‐190

GFD, gluten‐free diet; GIP, gluten immunogenic peptides.

a Conversion factor x = GIP (μg/g stool) to y = gluten daily consumption (mg) is y = 0.0649x^2^ + 1.0461x.

### 3.3 Correlation between faecal GIP and serum antibodies

**Figure 3:**
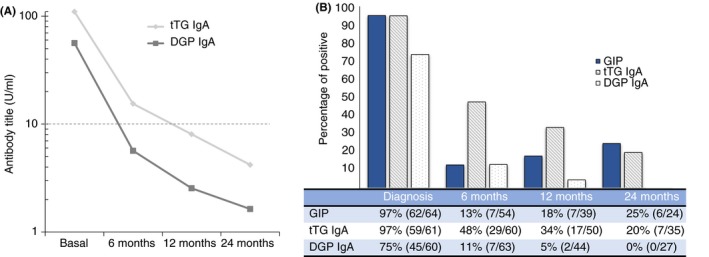
Evolution of GIP, tTG and DGP antibodies in patients with newly diagnosed coeliac disease. (A) tTG and DGP antibody levels vs time. (B) Percentage of dietary transgressions according GIP, tTG and DGP antibodies during the study period. DGP, deamidated gliadin peptide antibody; tTG, tissue transglutaminase antibody; GFD, gluten‐free diet; GIP, gluten immunogenic peptides. The cut‐off >10 U/mL

Serum tTG and DGP antibodies levels declined during follow‐up (Figure 3A). For tTG antibody, 48% were positive at the 6 months visit, 34% at 12 months and 20% at 24 months. For DGP antibody, positive results were obtained in 11% at 6 months, 5% at 12 months and 0% at 24 months. In contrast, rates of GIP positive stools showed an upward trend during the follow‐up period and were highest at the last visit (Figure 3B).

Considering the absolute values for both tTG and DGP antibodies at each visit, we did not find concordance for GIP, with very low kappa values, far from statistical significance (*P *>* *0.1). Nor was there statistical concordance when using dichotomous terms that is, positive when GIP were detected at least one follow‐up visit (6, 12, or 24 months), and negative when GIP were not detected during follow‐up (Table 3). The kappa values were very low, far from statistical significance (*P *>* *0.1) in all cases.

**Table 3:** Evolution of the GFD according to the comparative GIP and coeliac disease serologies

Time of GFD	Comparison GIP vs	λ_c_	95% CI λ_c_	Criterios azzimonti	Kappa (*P*)
Evolutionary	tTG IgA	57.4	42.3‐68.4	No	0.107 (0.342)
	DGP IgA	65.6	52.9‐78.0	No	0.103 (0.423)

CI, confidence interval; DGP, deamidated gliadin peptide antibody; GFD, gluten‐free diet; GIP, gluten immunogenic peptides; tTG, tissue transglutaminase antibody.

**Figure 4:**
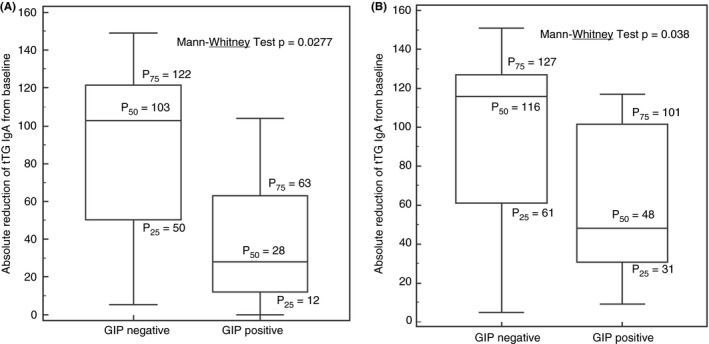
Absolute reduction of tTG antibody level at (A) 6 mo and (B) 12 mo in patients with detectable GIP and non GIP detected. GIP, gluten immunogenic peptides; tTG, tissue transglutaminase

Figure 3 displays the absolute reduction in the tTG antibody titer after 6 months (Figure 3A) and 12 months (Figure 3B) of GFD therapy. We also found a substantial, significant, reduction in the tTG antibody with respect to the basal (diagnosis) levels; this trend was stronger in the group of patients adhering to the GFD, as demonstrated by the negative GIP, with reductions of 103 U/mL (IQR 50‐122) vs 28 U/mL (IQR 12‐63) in the positive GIP patients after 6 months of GFD (*P *=* *0.028) (Figure 4A). This differential kinetic behaviour of the tTG antibody in the two groups was also observed at 12 months of treatment: 116 U/mL (IQR 61‐127) in adherents vs 48 U/mL (IQR 31‐101) (*P *=* *0.038) in non‐adherents (Figure 4B).

## 4 DISCUSSION

In this study of children with coeliac disease, the rate of GIP detection in stool dropped dramatically from 97% at diagnosis to 13% after 6 months of GFD. This high level of adherence after a GFD was not maintained on follow‐up. Some children may relax the GFD as evidenced by an increasing rate of GIP detection with time. We found that 46% of non‐adherent participants had two or more GIP positive stools on a GFD. This suggests a behavioural pattern involving repeated or chronic gluten exposure rather than infrequent episodic exposures. As expected, the stool GIP concentration in many non‐adherent patients was low, close to the limit of quantification (0.16 μg GIP per gram faeces). Nevertheless, this level of exposure is likely significant as children who had detectable GIP in their stool at any time after diagnosis had a more prolonged elevation of their serum tTG antibody than those with all stools being negative while trying to follow a GFD. Furthermore, some patients had faecal GIP levels similar to those of healthy controls on a gluten containing diet.^19^, ^20^

Investigation of factors underlying gluten exposure was beyond the scope of the current study; however, it must not be assumed that gluten ingestion was intentional. Although all families received GFD education from a dietician, it cannot be expected that they are implementing a GFD correctly. Similar to our previous studies,^20^ children younger than two years old had the highest and most sustained adherence to GFD using objective measures. This group, beside having overall a more controlled diet, is highly dependent on their parent or guardian to be fed; therefore, strict control over the diet may be easier to achieve compared with older children (7‐18 years old). Thus these differences may reflect the increasing autonomy of the patient regarding dietary decisions. Adolescence is a developmental phase characterised by rebellion and others have found that teenagers may be particularly susceptible to the burden of a GFD related to stigmatization and are more likely intentionally non‐adherent in such settings.^25^, ^26^, ^27^ Alternatively, older children and adolescents are also more likely to eat outside the home (eg, at school, at a friends’ home) thus having food prepared by persons not always well‐informed about the GFD requirements. The increased rate of GIP detection with age may simply reflect that these behaviours are inherently more “risky” with regards to gluten exposure. Among adults, symptomatic suspected gluten reactions are most commonly associated with eating at restaurants.^28^ Alternatively, it may be that the habit of GFD adherence, when established in early childhood, is maintained through adolescence and adulthood. This would suggest that diagnosing patients with coeliac disease as early in life as possible should be a priority to avoid social problems and physical and psychological deterioration.^29^

The initial symptomatic response to a GFD tends to be relatively rapid, occurring within days to weeks, especially in patients presenting with classical symptoms. Conversely, gluten exposure on a GFD may not evoke symptoms.^28^, ^30^ We found the rate of GIP positive stools increased with time since diagnosis, which may reflect a tendency to relax GFD adherence over time as patients identify that they may tolerate some gluten without symptoms and adjust their diet accordingly. The estimated gluten intake of patients on a GFD (median 0.104 grams per day) was be more than 30‐fold less than the typical amount used in gluten challenge (3‐10 g/d).^30^ Perhaps, ingestion of 0.1‐0.5 g gluten does not generate symptoms in a significant proportion of non‐adherent patients. A safe threshold for gluten consumption by persons with coeliac disease has not been established; however, it is accepted that gluten tolerance varies widely.^31^, ^32^ Consistent with this notion, those who were symptomatic at diagnosis were less likely than those who were asymptomatic to have detectable GIP in the stool. Other investigators have also identified a desire to avoid symptoms as a motivation for adhering strictly to a GFD.^33^, ^34^, ^35^

Our finding that most children were able to substantially reduce their gluten ingestion within 6 months has implications for the definition of “non‐responsive coeliac disease”. A categorization rather than a diagnosis, non‐responsive coeliac disease has been defined as “persistent symptoms, signs, or laboratory abnormalities typical of coeliac disease despite 6‐12 months of dietary gluten avoidance”.^36^ The duration of gluten avoidance required to be considered non‐responsive has been controversial, with some groups requiring at least 12 months of gluten restriction before considering patients non‐reponsive^37^, ^38^ and others suggesting that inadequate symptomatic response after 6 months on a GFD should prompt investigation for etiologies other than coeliac disease.^39^ Our data suggest that 6 months may be a reasonable definition as most children substantially reduced their gluten intake within this timeframe. Regardless of the definition, gluten ingestion (either intentional or inadvertent) is consistently found to be the leading cause of non‐responsive coeliac disease.^38^, ^39^ Testing for GIP in stool may be a useful tool in the evaluation of non‐responsive coeliac disease. Identification of ongoing gluten exposure may guide treatment and obviate the need for expensive and invasive investigations.

Our findings also help to clarify the interpretation of coeliac serology tests after a diagnosis of coeliac disease is established. Although widely used to monitor patients on a GFD and highly specific for persistent villous atrophy on a GFD, the low sensitivity of serum tTG, EMA and DGP antibodies tests renders a negative test substantially less informative.^16^, ^40^ We found that those with detectable levels of GIP in stool had a more prolonged elevation and a more gradual fall in tTG antibody than those whose stools tested negative. However, in absolute and evolutive (dichotomous) terms, no concordance is observed between the tTG antibody and GIP levels.

Strengths of our study include the use of a robust and objective measure of gluten exposure in a population of children who all received formal GFD education and who were followed prospectively from diagnosis. Contemporaneous food record completion and stool and serum collection allowed for accurate correlation of serology with faecal GIP detection. Loss to follow‐up is a limitation of our study that does complicate interpretation of increased gluten exposure.

In conclusion, serial analysis of faecal GIP allowed not only the first direct confirmation of gluten intake days before coeliac disease diagnosis, but also documentation of a substantial decrease in gluten consumption after diagnosis of coeliac disease and instruction in following a GFD. The introduction of GIP as an assessment tool of GFD adherence may help to ascertain dietary compliance and reduce the need for additional invasive investigations on follow‐up.

## AUTHORSHIP

*Guarantor of the article*: Carolina Sousa.

*Author contributions*: IC, VS, AC and CS performed the research; IC, VS, LO, BE, GC, JAG, CSi, AM, CRK, ER, ARH, JD, JAS, AC and CS collected and analysed the data; IC, VS, JAS, AC, CS designed the research study and wrote the paper; and IC, AC and CS contributed to the design of the study. All authors approved the final version of the manuscript.

## APPENDIX 1

### The authors’ complete affiliations

Isabel Comino, Verónica Segura, Carolina Sousa, Departamento de Microbiología y Parasitología, Facultad de Farmacia, Universidad de Sevilla, Sevilla, Spain; Luis Ortigosa, Hospital Universitario Nuestra Señora de La Candelaria, Tenerife, Spain; Beatríz Espín, Hospital Universitario Infantil Virgen del Rocío, Sevilla, Spain; Gemma Castillejo, Unitat Pediàtrica de Trastorns Relacionats amb el Gluten del Camp de Tarragona. IISPV, URV, Reus, Tarragona, Spain; José Antonio Garrote, Hospital Universitario Río Hortega, Valladolid, Spain; Carlos Sierra, Hospital Materno‐Infantil—Hospital Regional Universitario de Málaga, Málaga, Spain; Antonio Millán, Hospital Universitario Virgen de Valme, Sevilla, Spain; Carmen Ribes‐Koninckx, Hospital Universitario y Politécnico La Fe, Valencia, Spain; Enriqueta Román, Hospital Universitario Puerta del Hierro, Majadahonda, Madrid, Spain; Alfonso Rodríguez‐Herrera, Instituto Hispalense de Pediatría, Sevilla, Spain; Jacobo Díaz, Hospital Universitario de Ceuta, INGESA, Ceuta, Spain; Jocelyn Anne Silvester, University of Manitoba, Winnipeg, Manitoba, Canada; Boston Children's Hospital and Beth Israel Deaconess Medical Center, Boston, Massachusetts; Ángel Cebolla, Biomedal S.L., Sevilla, Spain.
